# Scoping Meta-Review of Methods Used to Assess Artificial Intelligence-Based Medical Devices for Heart Failure

**DOI:** 10.3390/bioengineering10101109

**Published:** 2023-09-22

**Authors:** Rossella Di Bidino, Davide Piaggio, Martina Andellini, Beatriz Merino-Barbancho, Laura Lopez-Perez, Tianhui Zhu, Zeeshan Raza, Melody Ni, Andra Morrison, Simone Borsci, Giuseppe Fico, Leandro Pecchia, Ernesto Iadanza

**Affiliations:** 1 Fondazione Policlinico Universitario Agostino Gemelli IRCCS—The Graduate School of Health Economics and Management (ALTEMS), 00168 Rome, Italy; 2 School of Engineering, University of Warwick, Coventry CV4 7AL, UK; davide.piaggio@warwick.ac.uk (D.P.); martina.andellini@opbg.net (M.A.); zeeshan-raza.raza@warwick.ac.uk (Z.R.); leandro.pecchia@unicampus.it (L.P.); 3 Life Supporting Technologies, Photonics Technology and Bioengineering Department, School of Telecommunication Engineering, Universidad Politécnica de Madrid, 28040 Madrid, Spainllopez@lst.tfo.upm.es (L.L.-P.); gfico@lst.tfo.upm.es (G.F.); 4 NIHR London In-Vitro Diagnostics Cooperative, Imperial College of London, London W2 1NY, UK; 5 Canadian Agency for Drugs and Technologies in Health, Ottawa, ON K1S 5S8, Canada; andra_morrison@hotmail.com; 6 Department of Learning, Data Analysis, and Technology, Cognition, Data and Education (CODE) Group, Faculty of Behavioural Management and Social Sciences, University of Twente, 7522 Enschede, The Netherlands; 7 School of Engineering, University Campus Bio-Medico, 00128 Rome, Italy; 8 International Federation of Medical and Biological Engineering, B-1090 Brussels, Belgium; 9 Department of Medical Biotechnologies, University of Siena, 53100 Siena, Italy

**Keywords:** artificial intelligence, machine learning, health technology assessment, heart failure, scoping review, decision making, value assessment

## Abstract

Artificial intelligence and machine learning (AI/ML) are playing increasingly important roles, permeating the field of medical devices (MDs). This rapid progress has not yet been matched by the Health Technology Assessment (HTA) process, which still needs to define a common methodology for assessing AI/ML-based MDs. To collect existing evidence from the literature about the methods used to assess AI-based MDs, with a specific focus on those used for the management of heart failure (HF), the International Federation of Medical and Biological Engineering (IFMBE) conducted a scoping meta-review. This manuscript presents the results of this search, which covered the period from January 1974 to October 2022. After careful independent screening, 21 reviews, mainly conducted in North America and Europe, were retained and included. Among the findings were that deep learning is the most commonly utilised method and that electronic health records and registries are among the most prevalent sources of data for AI/ML algorithms. Out of the 21 included reviews, 19 focused on risk prediction and/or the early diagnosis of HF. Furthermore, 10 reviews provided evidence of the impact on the incidence/progression of HF, and 13 on the length of stay. From an HTA perspective, the main areas requiring improvement are the quality assessment of studies on AI/ML (included in 11 out of 21 reviews) and their data sources, as well as the definition of the criteria used to assess the selection of the most appropriate AI/ML algorithm.

## 1. Introduction

Heart failure (HF) is a multi-faceted and life-threatening syndrome and is one of the leading causes of mortality and hospitalisation. According to statistics, 64.3 million people suffered from HF globally in 2017 [1,2]. The most recent definition describes HF as a clinical syndrome with symptoms and/or signs caused by a structural and/or functional cardiac abnormality, corroborated by elevated natriuretic peptide levels and/or objective evidence of pulmonary or systemic congestion [3]. HF patients usually undergo numerous diagnostic tests, procedures, and therapies that generate a large amount of data. These data have been used in recent decades to train algorithms and develop artificial intelligence and machine learning (AI/ML) applications for different purposes, ranging from the identification of risk factors for incident HF to disease classification, early diagnosis, early detection of decompensation, risk stratification, management, and the organisation of health services, among others [4,5,6,7].

The widespread use of AI/ML solutions is expected to drastically change the domain of medicine and healthcare systems, especially after the World Health Organisation (WHO) included AI/ML-based medical devices (MDs) in the definition of “Health technology” [8,9]. As the implementation, adoption, and use of AI/ML MDs in healthcare settings are crucial from several points of view (legal, ethical, social, economic, and organisational aspects), their worth must be assessed using approaches, even if modified, that are similar to those used to assess the value of other medical innovations.

Some barriers to the implementation of accurate AI/ML MDs for HF are already known. For instance, it is often difficult to identify a population with high enough event rates to demonstrate the effects of the solution [4]. Such a problem of representativeness in trials is usually regarded as a methodological issue in both the quality of the data collected and the study design. This issue, when identified, is often partially addressed by meticulously specifying the inclusion criteria of participant selection [10]. On top of methodological issues, another problem associated with AI solutions in the safety-critical setting of HF is the lack of accurate confidence intervals for predictions. This is a potentially serious issue that can affect the trustworthiness and robustness of AI solutions, as well as the generalizability of the results [4,11]. There are ongoing research efforts to maximise the accuracy of the confidence intervals of AI/ML MD solutions [12] and optimise these solutions. Certainly, agreement on a common approach to assess and judge the quality of these systems is missing.

For health technologies, this process is normally overseen by Health Technology Assessment (HTA) [13] principles and criteria. At the international level, healthcare experts are trying to map and identify the key challenges (e.g., regulatory, ethical, etc.) involved in assessing AI in the real world, and reach a consensus on HTA methods and frameworks used to assess the quality of AI applications [14]. However, although specific HTA frameworks for diagnostic technologies, medical and surgical interventions, and screening technologies are publicly available, frameworks for telemedicine or mobile health [15,16] have only recently been developed (e.g., the MAST-AI (Model for Assessing the value of Artificial Intelligence in medical imaging) [17]). Moreover, even if HTA agencies, such as the National Institute for Health and Care Excellence (NICE) in the UK, started defining standard frameworks for digital technologies [16], there is currently no agreement on a common HTA framework for the specific assessment of different types of AI/ML-based MDs.

The other aim of this manuscript is to explore and systematise all the methods available in the literature that have been exploited by healthcare professionals to assess the quality of AI-based MDs, specifically those related to heart failure (HF).

## 2. Methods

The International Federation of Medical and Biological Engineering (IFMBE) created a multidisciplinary working group to discuss potential methods for assessing AI/ML-based medical devices. The group was composed of 15 expert professionals in biomedical engineering, human factors, health economics, and the HTA (with more than two years of expertise). A series of focus groups were organised to establish the research question and the inclusion and exclusion criteria, as well as come to an agreement on various definitions, as reported in Box 1.

Box 1 **Glossary.**

**AI/ML-based medical devices (MDs)**

 

Medical device software that includes AI/ML algorithms.

 

**Artificial Intelligence (AI)**

AI is broadly defined as the science and engineering of making intelligent machines, especially intelligent computer programs [18].

 

**Health technology**

Health technology is an intervention developed to prevent, diagnose, or treat medical conditions; promote health; provide rehabilitation; and organise healthcare delivery. The intervention can be a test, device, medicine, vaccine, procedure, program, or system [13].

 

**Health Technology Assessment (HTA)**

HTA is a multidisciplinary process that uses explicit methods to determine the value of a specific health technology at different points in its lifecycle. The purpose is to inform decision making to promote an equitable, efficient, and high-quality health system [13].

 

**Heart failure (HF)**

A clinical syndrome with symptoms and/or signs caused by a structural and/or functional cardiac abnormality, corroborated by elevated natriuretic peptide levels and/or objective evidence of pulmonary or systemic congestion [3]. The definition of heart failure encompasses acute coronary syndromes and atrial fibrillation.

 

**HTA framework**

A methodological framework for the production and sharing of HTA information based on a standardised set of HTA questions (the ontology) that allows users to define their specific research questions within a hierarchical structure. (Definition adapted from EUnetHTA Core Model® [19]).

 

**Machine learning (ML)**

ML, a branch of artificial intelligence (AI) and computer science, focuses on developing systems that can learn and adapt without following explicit instructions, imitating the way humans learn. It gradually improves its accuracy by using algorithms and statistical models to analyse and draw inferences from patterns in data [20].

 

**Medical device software (MDSW)**

Medical device software is software that is intended to be used alone or in combination for a purpose specified in the definition of a “medical device” in the medical devices regulation (Article 2(1) of Regulation (EU) 2017/745—MDR) or in vitro diagnostic medical devices regulation (Article 2(2) of Regulation (EU) 2017/746) [21].

The defined research question is as follows: “What are the methods used and evidence collected to assess AI/ML-based medical devices for heart failure and what are their strengths and limitations?”

A meta-review [22] of systematic reviews, scoping reviews, and meta-analyses was conducted, which focused on the AI/ML algorithms developed for and used in the management of adult patients with heart failure, with a particular focus on HTA techniques and methods used, if any. The meta-review was conducted in line with the extended version of the Preferred Reporting Items for Systematic Reviews and Meta-Analyses (PRISMA-ScR) guidelines [23,24].

Embase and Scopus were searched for relevant literature. In addition, grey literature published on major HTA agencies’ websites was included. The literature search covered the period from January 1974 to October 2022. The detailed search string is reported in Appendixes Appendix A.1 and Appendix A.2.

### 2.1. PICO and Eligibility Criteria

The PICO (Population, Intervention, Comparator, Outcomes) elements used in our review were as follows: Population—patients affected by HF; Intervention—AI/ML-based MDs; Comparator—traditional methods used in clinical practice and conventional statistical methods; Outcomes—accuracy, effectiveness, and organisational outcomes such as admissions/readmissions and/or impact on the length of stay (LOS).

The inclusion and exclusion criteria were based on the publication type and topic. Only studies reporting on AI/ML methods applied to the prediction of HF risk, monitoring, and management of the disease were included. No limitation was considered for the setting of their use (e.g., inpatient, outpatient, community). In addition, only systematic or scoping reviews or meta-analyses were considered for inclusion. All the other publication types, as well as all those out of our scope, were excluded.

### 2.2. Identification and Screening

The titles and abstracts of the retrieved articles and their full texts were screened by two researchers independently. Any conflict between the reviewers was resolved by the involvement of a third independent reviewer.

### 2.3. Data Extraction and Analysis

For the data extraction, an ad hoc table was created to collect data on both the review and the AI/ML methodology, including data sources (literature database), quality assessment, comparison of results, and clinical and organisational endpoints.

The included studies were categorised as (i) ‘meta analysis’, (ii) ‘systematic review’, or (iii) ‘narrative review’.

To manage the data/evidence traceability, from each of the selected studies, we extracted information regarding the specific literature search engines and the countries of the items included in the review.

The AI/ML algorithms were categorised using the framework adopted by Graili et al. [25] and proposed by Brownlee [26]. The algorithms were categorised based on their function or form. The AI/ML framework includes more than 60 algorithms and divides them into 12 types: deep learning, ensemble models, neural networks, regulation, rule system, regression, Bayesian, decision trees, dimensionality reduction, instance-based, and clustering.

Many guidelines have been proposed for reporting trials that evaluate AI-driven technologies (i.e., TRIPOD-AI [27], STARD-AI [28], SPIRIT-AI [11], CONSORT-AI [29], and DECIDE-AI [30]). They differ in many aspects, including the stage of development of the technology and the study design. We recorded information regarding which guidelines, if any, had been adopted in the selected papers. It was considered a proxy of the quality in terms of the attention paid by the authors to the appraisal of the studies.

Since the EUnetHTA guidelines [31] mention the importance of identifying the appropriate comparator(s) in assessments, we also considered whether the studies included in our review explicitly defined the comparators.

Finally, we developed a comprehensive overview and synthesis of the evidence, without focusing on each study included in each review.

## 3. Results

**Figure 1:**
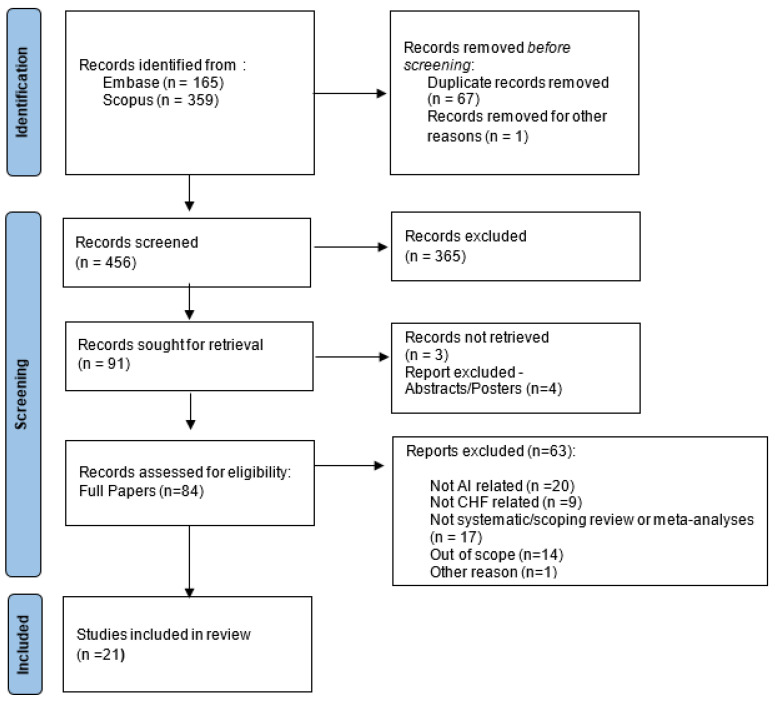
Screening and selection of papers included in the scoping meta-review.

The scoping review identified 524 potentially relevant papers. After removing duplicates, 456 underwent title and abstract selection; 365 articles were excluded, as the items did not match the eligibility criteria. A full-text review was conducted for 84 articles; 61 articles were excluded, as they were not related to AI or CHF or did not meet our inclusion criteria. Overall, 21 reviewed studies met our inclusion criteria and were included in our analysis. The PRISMA flow diagram (Figure 1) reports this process and the reasons for exclusion at each stage.

### 3.1. Selected Papers

As shown in Table 1, out of 21 studies, 4 reported the results of a meta-analysis, 11 were systematic reviews, 1 was a scoping review, and 5 were narrative reviews. The reviews included in the meta-review discussed and summarised data from a mean of 49 studies, spanning the five articles presented by Grün et al. [32] to the list of 122 studies presented by Bazoukis et al. [33]. The earliest records available were published in 2018, whereas the latest items were published in 2022.

**Figure 2:**
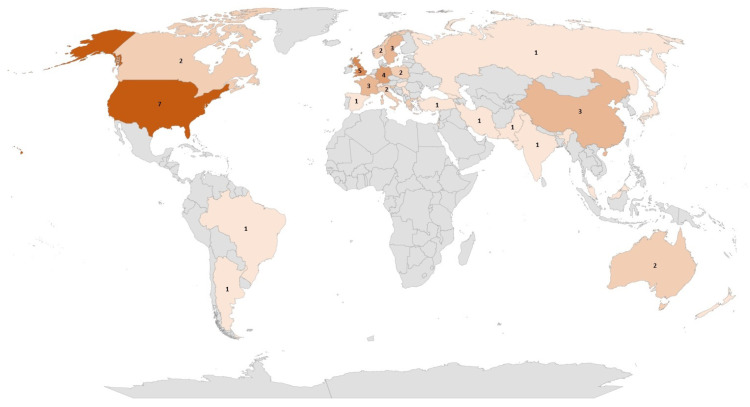
Countries for which at least one study was included in the articles considered in the scoping review.

The data sources of the selected reviews were quite diverse. The most frequently adopted sources for item identification and selection were Medline (in 11 out of 21 studies), Pubmed (n = 8), Cochrane Library (n = 6), Embase (n = 5), and Web of Science (n = 5). In terms of geographical representation (Figure 2), the selected articles included studies conducted mainly in North America (seven in the US and two in Canada) and Europe (mainly in the United Kingdom (n = 5), Germany (n = 4), and the Netherlands (n = 4)). Only a few studies were conducted in Asia (China (n = 3) and Korea (n = 2)) and Australia (n = 3).

The majority of reviews (16 out of 21) aimed to provide an overview of methods and AI/ML models developed for HF [32,33,35,36,37,38,41,43,44,45,46,47,48,49,50,51]. Eight reviews [33,34,35,37,39,42,43,44] explicitly analysed the performance of the AI/ML algorithms. Eight reviews included additional goals, such as current utilisation of and barriers to the diffusion of AI/ML in clinical practice, and future developments [38,40,41,42,45,46,51,52]. In one review, the aim was also to provide some tools or hints for evaluating the quality of studies on AI/ML applications for HF [42].

### 3.2. Clinical Aspects

**Figure 3:**
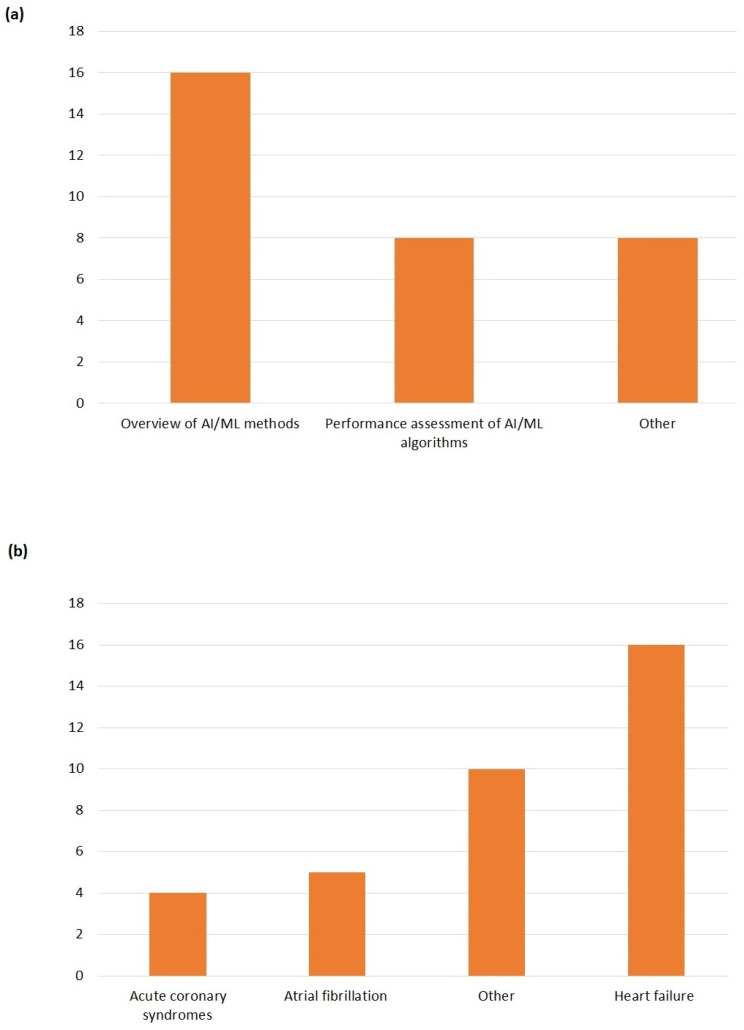
(**a**) Aims of selected studies. (**b**) Clinical indications.

We adopted quite a vast definition of HF (as reported in Box 1), and some studies covered more than one clinical indication. Sixteen out of 21 papers (Figure 3) focused on (congestive) heart failure. Five studies also considered atrial fibrillation, whereas four articles included acute coronary syndromes. Quite common (as indicated by the Others category in Figure 3) was the inclusion under the HF umbrella of other clinical conditions such as cardiovascular diseases (CVD), coronary artery disease (CAD), ischemic heart diseases, stroke, and valvular heart diseases.

In terms of clinical application, the majority of cases focused on risk prediction and/or early diagnosis (n = 19). The classification of HF was considered in only seven cases, and the prognosis was included in only five cases. In terms of the clinical setting making use of AI/ML algorithms, in half of the cases, both the inpatients and outpatients were considered. Ten studies did not report this information.

### 3.3. AI/ML Algorithms

**Figure 4:**
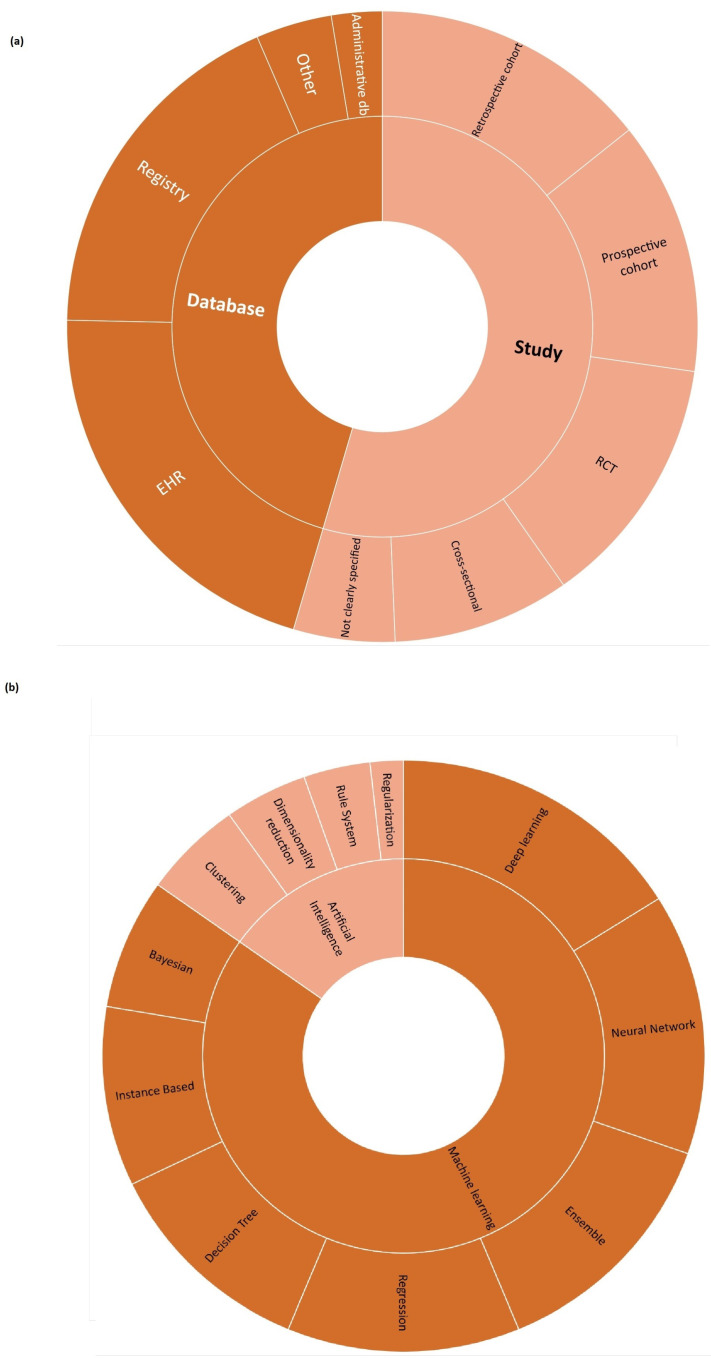
(**a**) Datasets. (**b**) Methods.

The datasets used as the base of AI/ML algorithms were different (Figure 4, Table A1). Among the databases, electronic health records (EHR, n = 16) and registries (n = 14) were the preferred ones. Among the studies, retrospective cohorts (n = 11), prospective cohorts (n = 10), and randomised controlled trials (RCT, n = 10) were the most common data sources. In three cases, other data sources were adopted (electrocardiogram datasets, claims data). In four of the selected papers, all or one of the AI/ML datasets were not clearly specified.

To investigate the AI algorithm used category, the same framework reported in [25] was adopted. According to our scoping review (Figure 4, Table A2), deep learning was the most common type of algorithm used (reported in 18 papers), followed by neural networks (n = 16), ensemble (n = 15), and regression techniques (n = 14).

Finally, in terms of the quality assessment of the studies included in the selected reviews, in 11 cases ([33,34,35,36,37,39,41,42,43,44,47]), the quality assessment activity was clearly mentioned but no homogeneity emerged in terms of the adopted methods. Ad hoc approaches or the adaptation of available tools were also employed [34,41,42]. No preference emerged towards TRIPOD-AI or any other reporting guidelines. In five reviews, the risk of bias was addressed using validated tools, such as PROBAST (Prediction model Risk Of Bias ASsessment Tool) [35,46] or QUADAS-2 (Quality Assessment of Diagnostic Accuracy Studies 2) [36,39,43]. Other reported scales included AI-TREE, CHARMS, AHA, PROGRESS, and the Critical Appraisal Skills Program (CASP) checklist.

### 3.4. Performance, Effectiveness, and Safety

Great attention was paid to the assessment of test performance or the accuracy of AI/ML algorithms. In 11 reviews, AUC/ROC or sensitivity/sensibility were explicitly reported. In addition, the benefit–risk profile was investigated, with more than half of the selected reviews (n = 12) paying great attention to the impact on mortality. The impact on the incidence of progression of HF was assessed in 10 reviews, whereas the impact on admission/readmissions or LOS was reported in 13 cases. In four publications, it was unclear how efficacy or effectiveness had been investigated.

**Figure 5:**
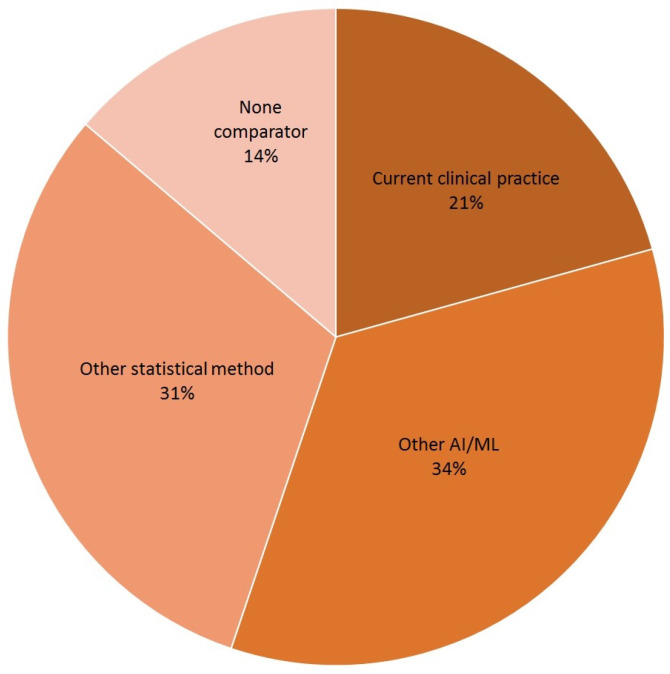
Comparators of AI/ML algorithm-based MDs.

Finally, the HTA requires a comparative assessment. EUnetHTA specifies that the comparator should be the alternative intervention(s) against which the intervention under assessment should be compared. As shown in Figure 5, current clinical practice was included in six cases (21 per cent). Comparisons with other AI/ML algorithms or reporting the results of conventional statistical methods were more common.

## 4. Discussion

The main finding of our scoping review is that when it comes to AI/ML models for HF for different purposes (e.g., identification of risk factors, disease classification, early diagnosis, etc.), there is a heterogeneous set of approaches adopted by developers in terms of not only algorithm design but also evidence generated and reported on the characteristics and performance of these algorithms. Such heterogeneity became clear when considering the aims of the selected studies, as well as the methods and data sources of the AI/ML algorithms, and identifying the comparators.

Our analysis mainly focused on the current types of evidence available in the assessment of AI/ML-based MDs.

We did not discuss the details of the accuracy of AI/ML algorithms or the likelihood of transition of AI/ML algorithms into clinical practice. In the same way, we did not investigate the factors affecting the choice of quality assessment scales. Various quality assessment scales are available for appraising the quality of clinical research related to AI/ML technologies, but there seems to be no unified standard for choosing those scales.

### 4.1. Key Findings

The HTA is based on the available evidence for assessing a health technology in comparison with current clinical practice [13]. Considering the evidence collected on AI-based MDs for HF, some common gaps have emerged. They are:

- Generalisability and representativeness. The majority of the retrieved systematic reviews mainly considered cases in developed countries (Figure 2), with an associated risk of discrimination and lack of representativeness. From an HTA perspective, this has consequences on the generalisability of both the trial results to other geographical areas and the performance of AI/ML algorithms to other populations of patients, not included in the data sources employed to develop the algorithms. This limits not only the recommendation an HTA can provide but also its transferability to other settings [53].
- Quality of available evidence. Guidelines for reporting trials that evaluate interventions are increasingly used when it comes to modelling the impact of AI-driven technologies. For instance, TRIPOD-AI [27] was developed to predict models, STARD-AI [28] was developed for diagnostic accuracy studies, and SPIRIT-AI [11] and CONSORT-AI [29] were developed for randomised controlled studies. Recently, the Developmental and Exploratory Clinical Investigations of DEcision support systems driven by Artificial Intelligence (DECIDE-AI) approach [30] was proposed. This approach aims to improve the reporting of early-stage clinical evaluations of AI-based technologies, independently of the study design chosen. We encountered both a lack of attention and variability in reporting quality assessment in reviews on AI/ML algorithms for HF, as well as a lack of agreement on which criteria/scale should be adopted to investigate quality. This is in line with the observation by Shazad et al. [54], who highlighted that the quality of reporting of randomised controlled trials in AI is suboptimal. It is also in line with the finding of Plana et al. [55], who reported high variability in adherence to reporting standards. At the same time, available tools adapted to AI are not yet fully able to capture the peculiarities of AI/ML algorithms and trials. As an immediate consequence, practitioners should interpret with caution the findings of studies regarding AI/ML algorithms for HF.
- AI/ML methods. Different models are currently being developed to manage HF, but no guidelines are available for assessors to investigate in detail the reliability of each algorithm and capture the added value of one AI/ML model in comparison to others. Given the long list of methods currently used, as shown in Figure 4, the HTA is neither able to select the most appropriate comparators nor conduct a comparative assessment of AI/ML algorithms.
- Comparative evidence. Only a small proportion of studies evaluated AI/ML algorithms without conducting any kind of comparison. This is a promising result (Figure 5). However, the preferred comparator was not current clinical practice, as requested by the HTA, but rather other AI/ML models or other statistical methods. As occurs with any expected disruptive technology, the choice of the comparator is not easy. It is not just a new active principle or MD, AI/ML promises to be a new paradigm, able to redefine clinical pathways. In this case, direct or indirect comparisons with current clinical practice are even more important and necessary.
- Data sources. Last but not least, the data at the core of AI/ML algorithms are crucial. They are usually real-world data/evidence (RWD/RWE), which are becoming more and more relevant for the HTA and decision makers. While investigating the complexity of AI for the HTA, Alami et al. [14] mentioned not only data quality and representativeness but also fragmented and unstructured data coming from different sources. It becomes clear how that adds complexity to a scenario where the role of RWD/RWE and issues such as real-world data availability, governance, and quality are not fully addressed [56].

### 4.2. Strengths and Limitations

This study systematically analysed and synthesised data from multiple studies, providing a more robust and reliable analysis compared to a single study. We investigated whether AI/ML research and development is relevant to the HTA. In a way, our analysis integrates the analysis carried out by Sharma et al. [57], which demonstrates a dissonance between research and practice. The HTA plays an intermediate role in the flow from research to clinical practice. This study, therefore, provides an overview of the different methods used to evaluate artificial intelligence-based medical devices for heart failure. It covers various aspects, such as data collection, analysis, and evaluation of existing articles, providing readers with a comprehensive understanding of the topic and suggesting that the lack of relevant evidence for the HTA could impact market access and adoption. The insights gleaned from this study are highly applicable to medical professionals and researchers involved in the development and evaluation of AI-based MDs for heart failure and can potentially serve as a reference for those seeking to improve their evaluation methods.

However, due to the relatively new nature of AI-based MDs for HF, the data available for review are limited. This limitation affects the completeness of the analysis and the conclusions drawn from the study. The quality of the data extracted from the articles included may vary. The inclusion of low-quality studies may affect the overall conclusions drawn from the review, as comprehensive control criteria were not established. In addition, the methods used to evaluate artificial intelligence-based medical devices for heart failure may vary depending on the device, data, and intended use. Therefore, our study does not cover all areas of the HTA. Ethical and legal aspects are completely outside our scope. Finally, to ensure that the selection process is as objective as possible, the lack of standardisation of the methods and reporting needs to be better investigated.

### 4.3. Further Development

This scoping review allowed us to identify the challenges posed by AI/ML-based MDs for a specific clinical condition (HF) and from the perspective of the HTA. The situation is rapidly evolving, as demonstrated by the significant increase in the number of studies on AI/ML algorithms and their implementation in clinical practice [58]. Therefore, although our analysis requires an update in three to five years, it is a useful starting point to investigate more aspects in detail, such as the quality and representativeness of data sources, as well as the criteria used to select the comparators.

## 5. Conclusions

In our scoping meta-review of the methods used to assess artificial intelligence (AI)-based medical devices (MDs) for heart failure (HF), we uncovered critical insights into the dynamic landscape of AI applications in healthcare. Our analysis emphasised the heterogeneity in the approaches taken by developers, highlighting the diversity in AI/ML models designed for various HF management purposes. This diversity extends beyond algorithms to encompass evidence generation and reporting, signifying the evolving nature of this field.

We identified key challenges that warrant attention in the evaluation of AI-based MDs for HF. Notably, the limited generalisability of the evidence due to a predominant focus on developed countries poses a barrier to making recommendations applicable to diverse healthcare settings. Additionally, the absence of standardised quality assessment practices for AI/ML in clinical research raises concerns about result interpretation. It is crucial to develop and agree on reporting standards and assessment tools tailored to the unique features of AI/ML technologies.

The proliferation of AI/ML methods presents both promise and complexity. The absence of guidelines for assessing reliability and value in these methods complicates comparative assessments, hindering the ability to select the appropriate comparators and conduct thorough evaluations. Moreover, our findings reveal a shift in comparison practices, with AI/ML algorithms often benchmarked against each other or other statistical methods rather than current clinical practice. This departure from standard health technology assessment (HTA) practices underscores the need for comprehensive comparative evidence.

Real-world data/evidence (RWD/RWE) emerged as a vital consideration, with its use becoming increasingly relevant to the HTA and decision makers. However, the challenges associated with RWD/RWE, such as data quality, representativeness, and fragmentation, amplify the complexity of AI/ML evaluation. Addressing these challenges will be pivotal for harnessing the potential of AI in HF management.

In conclusion, our meta-review bridges the gap between AI/ML research and clinical practice, offering a comprehensive overview of AI-based MDs for HF evaluation methods. Although our study has strengths, including systematic analysis and an emphasis on the HTA’s intermediate role, it also has limitations due to the evolving nature of AI applications and the variability in the data and methods used.

Looking forward, we recommend revisiting this analysis in three to five years to check the progress and the emerging challenges. Future research should delve deeper into aspects like data quality, representativeness, and the criteria used to select the appropriate comparators.

In summary, AI-based MDs hold promise for enhancing HF management, but assessing them poses multi-faceted challenges. Our meta-review underscores the need for standardised evaluation practices, greater attention to data quality, and the pursuit of comprehensive comparative evidence. As AI/ML technologies continue to evolve, so too must our evaluation methods to ensure their safe and effective integration into clinical practice.

## Figures and Tables

**Table 1:** Summary of papers included in the scoping meta-review.

Type	Study ID	Citations *	Years Covered	No. Studies	Clinical Indication **
Meta-analysis	Gruen et al., 2020 [32]	10	2017–2020	5	HF
	Krittanawong et al., 2020 [34]	78	1966–2019	55	HF. ACS
	Nadarajah et al., 2021 [35]	2	Till March 2021	11	HF, AI, stroke
	Lee et al., 2022 [36]	6	1970-2021	102	HF, AI, Other
Systematic reviews	Mahajan et al., 2018 [37]	40	1948–2018	25	HF
	Medic et al., 2019 [38]	25	2013–2018	20	HF
	Banerjee et al., 2021 [39]	17	2000–2019	97	HF, ACS, AF
	Bazoukis et al., 2021 [33]	31	2005–2019	122	HF
	Mpanya et al., 2021 [40]	4	1993–2007	30	HF
	Reading Turchioe et al., 2021 [41]	4	2015–2020	37	HF, ACS, Other
	Shin et al., 2021 [42]	35	2000–2020	20	HF
	Wu et al., 2021 [43]	0	2015–2021	38	HF, Other
	Blaziak et al., 2022 [44]	1	Till March 2022	9	HF, ACS, AF
	Javeed et al., 2022 [45]	9	1995–2021	105	HF, other
	Sun et al., 2022 [46]	2	2010–2021	116	HF
Scoping reviews	Sun et al., 2022 [47]	0	Till December 2021	47	HF, Other
Narrative reviews	Tripoliti et al., 2017 [48]	167	2000–2017	N/A	HF
	Safdar et al., 2018 [49]	96	Till–2015	20	HF, Other
	Kilic, 2020 [50]	74	until 2019	N/A	HF, Other
	Maurya et al., 2021 [51]	1	N/A	N/A	HF
	Shu et al., 2021 [52]	1	N/A	16	HF, Other

* Citations: Citations in Google Scholar (until March 2023); ** Clinical indications: HF—heart failure; ACS—acute coronary syndromes; AF—atrial fibrillation

## Abbreviations

The following abbreviations are used in this manuscript:

AI/ML	Artificial intelligence/machine learning
AUC/ROC	Area under the ROC curve
EHR	Electronic Health Records
HF	Heart failure
HTA	Health Technology Assessment
MD	Medical device
MDSW	Medical device software
RCT	Randomised controlled trial

## Appendix A

### Appendix A.1. Search String—Embase

1 “heart failure”/
2 cardiomyopathy, dilated/
3 shock, cardiogenic/
4 exp ventricular dysfunction/
5 cardiac output, low/
6 ((heart or cardiac or coronary or myocardial) adj2 (failure or decompensation or death or incompetence or insufficiency)).ti,ab.
7 ((dilated or congestive) adj2 cardiomyopath*).ti,ab.
8 cardiogenic shock.ti,ab.
9 ((ventricular or ventricle*) adj2 (failure or insufficien* or dysfunction*)).ti,ab.
10 ((left ventricular or left ventricle) adj2 (failure or insufficien* or dysfunction*)).ti,ab.
11 lvsd.ti,ab.
12 scd.ti,ab.
13 scd.ti,ab.
14 hf.ti,ab.
15 chf.ti,ab.
16 or/1–15
17 artificial intelligence/
18 model,neural network/
19 models, neural network/
20 neural network model/
21 neural network models/
22 neural networks computer/
23 neural network computer/
24 Computational Intelligence/
25 Natural Language Processing/
26 (deep learn* or machine learn* or continuous learn*).ti,ab.
27 “neural network*”.ti,ab.
28 ((Artificial or Comput* or Machine) adj1 Intelligence).ti,ab.
29 Natural Language Processing*.ti,ab.
30 Computer Vision*.ti,ab.
31 or/17–30
32 16 and 31
33 (systematic review or meta-analysis).pt.
34 meta-analysis/ or systematic review/ or systematic reviews as topic/ or meta-analysis as topic/ or “meta analysis (topic)”/ or “systematic review (topic)”/ or exp technology assessment, biomedical/ or network meta-analysis/
35 ((systematic* adj3 (review* or overview*)) or (methodologic* adj3 (review* or overview*))). ti,ab,kf,kw.
36 ((quantitative adj3 (review* or overview* or synthes*)) or (research adj3 (integrati* or overview*))).ti,ab,kf,kw.
37 ((integrative adj3 (review* or overview*)) or (collaborative adj3 (review* or overview*)) or (pool* adj3 analy*)).ti,ab,kf,kw.
38 (data synthes* or data extraction* or data abstraction*).ti,ab,kf,kw.
39 (handsearch* or hand search*).ti,ab,kf,kw.
40 (mantel haenszel or peto or der simonian or dersimonian or fixed effect* or latin square*).ti,ab,kf,kw.
41 (met analy* or metanaly* or technology assessment* or HTA or HTAs or technology overview* or technology appraisal*).ti,ab,kf,kw.
42 (meta regression* or metaregression*).ti,ab,kf,kw.
43 (meta-analy* or metaanaly* or systematic review* or biomedical technology assessment* or bio-medical technology assessment*).mp,hw.
44 (medline or cochrane or pubmed or medlars or embase or cinahl).ti,ab,hw.
45 (cochrane or (health adj2 technology assessment) or evidence report).jw.
46 (comparative adj3 (efficacy or effectiveness)).ti,ab,kf,kw.
47 (outcomes research or relative effectiveness).ti,ab,kf,kw.
48 ((indirect or indirect treatment or mixed-treatment or bayesian) adj3 comparison*).ti,ab,kf,kw.
49 [(meta-analysis or systematic review).md.]
50 (multi* adj3 treatment adj3 comparison*).ti,ab,kf,kw.
51 (mixed adj3 treatment adj3 (meta-analy* or metaanaly*)).ti,ab,kf,kw.
52 umbrella review*.ti,ab,kf,kw.
53 (multi* adj2 paramet* adj2 evidence adj2 synthesis).ti,ab,kw,kf.
54 (multiparamet* adj2 evidence adj2 synthesis).ti,ab,kw,kf.
55 (multi-paramet* adj2 evidence adj2 synthesis).ti,ab,kw,kf.
56 or/33–55
57 56 and 32
58 remove duplicates from 57
59 limit 58 to yr=”2015 -Current”

### Appendix A.2. Search String—Scopus

1 TITLE-ABS-KEY (“heart failure” OR “Cardiac Failure” OR “Heart Decompensation” OR “Decompensation, Heart” OR “Heart Failure, Right-Sided” OR “Heart Failure, Right Sided” OR “Right-Sided Heart Failure” OR “Right Sided Heart Failure” OR “Myocardial Failure” OR “Congestive Heart Failure” OR “Heart Failure, Congestive” OR “Heart Failure, Left-Sided” OR “Heart Failure, Left Sided” OR “Left-Sided Heart Failure” OR “Left Sided Heart Failure”)
2 TITLE-ABS-KEY (“cardiomyopathy, dilated” OR “Cardiomyopathies, Dilated” OR “Dilated Cardiomyopathies” OR “Dilated Cardiomyopathy” OR “Cardiomyopathy, Familial Idiopathic” OR “Cardiomyopathies, Familial Idiopathic” OR “Familial Idiopathic Cardiomyopathies” OR “Familial Idiopathic Cardiomyopathy” OR “Idiopathic Cardiomyopathies, Familial” OR “Idiopathic Cardiomyopathy, Familial” OR “Congestive Cardiomyopathy” OR “Cardiomyopathies, Congestive” OR “Congestive Cardiomyopathies” OR “Cardiomyopathy, Congestive” OR “Cardiomyopathy, Idiopathic Dilated” OR “Cardiomyopathies, Idiopathic Dilated” OR “Dilated Cardiomyopathies, Idiopathic” OR “Dilated Cardiomyopathy, Idiopathic” OR “Idiopathic Dilated Cardiomyopathies” OR “Idiopathic Dilated Cardiomyopathy” OR “Cardiomyopathy, Dilated, LMNA” OR “Cardiomyopathy, Dilated, Autosomal Recessive” OR “Cardiomyopathy, Dilated, 1a” OR “Cardiomyopathy, Dilated, With Conduction Defect 1” OR “Cardiomyopathy, Dilated, with Conduction Deffect1” OR “Cardiomyopathy, Dilated, CMD1A”)
3 TITLE-ABS-KEY (“shock, cardiogenic” OR “Cardiogenic Shock”)
4 TITLE-ABS-KEY (“ventricular dysfunction” OR “Dysfunction, Ventricular” OR “Dysfunctions, Ventricular” OR “Ventricular Dysfunctions” OR “Right Ventricular Dysfunction” OR “Dysfunction, Right Ventricular” OR “Dysfunctions, Right Ventricular” OR “Right Ventricular Dysfunctions” OR “Ventricular Dysfunctions, Right” OR “Left Ventricular Dysfunction” OR “Dysfunction, Left Ventricular” OR “Dysfunctions, Left Ventricular” OR “Left Ventricular Dysfunctions” OR “Ventricular Dysfunctions, Left”)
5 TITLE-ABS-KEY (“cardiac output, low” OR “Output, Low Cardiac” OR “Low Cardiac Output” OR “Low Cardiac Output Syndrome”)
6 TITLE-ABS ((heart or cardiac or coronary or myocardial) W/2 (failure or decompensation or death or incompetence or insufficiency))
7 TITLE-ABS ((dilated or congestive) W/2 cardiomyopath!)
8 TITLE-ABS (“cardiogenic shock”)
9 TITLE-ABS ((ventricular or ventricle!) W/2 (failure or insufficien! or dysfunction!))
10 TITLE-ABS ((“left ventricular” or “left ventricle”) W/2 (failure or insufficien! or dysfunction!))
11 TITLE-ABS (lvsd)
12 TITLE-ABS (scd)
13 TITLE-ABS (scd)
14 TITLE-ABS (hf)
15 TITLE-ABS (chf)
16 or/1–15
17 TITLE-ABS-KEY (“artificial intelligence” OR “Intelligence, Artificial” OR “Computational Intelligence” OR “Intelligence, Computational” OR “Machine Intelligence” OR “Intelligence, Machine” OR “Computer Reasoning” OR “Reasoning, Computer” OR “AI (Artificial Intelligence)” OR “Computer Vision Systems” OR “Computer Vision System” OR “System, Computer Vision” OR “Systems, Computer Vision” OR “Vision System, Computer” OR “Vision Systems, Computer” OR “Knowledge Acquisition (Computer)” OR “Acquisition, Knowledge (Computer)” OR “Knowledge Representation (Computer)” OR “Knowledge Representations (Computer)” OR “ Representation, Knowledge (Computer)”)
18 TITLE-ABS-KEY (“model,neural network” OR “Computer Neural Network” OR “Computer Neural Networks” OR “Network, Computer Neural” OR “Networks, Computer Neural” OR “Neural Network, Computer” OR “Models, Neural Network” OR “Model, Neural Network” OR “Network Model, Neural” OR “Network Models, Neural” OR “Neural Network Model” OR “Neural Network Models” OR “Computational Neural Networks” OR “Computational Neural Network” OR “Network, Computational Neural” OR “Networks, Computational Neural” OR “Neural Network, Computational” OR “Neural Networks, Computational” OR “Perceptrons” OR “Perceptron” OR “Connectionist Models” OR “Connectionist Model” OR “Model, Connectionist” OR “Models, Connectionist” OR “Neural Networks (Computer)” OR “Network, Neural (Computer)” OR “ Networks, Neural (Computer)” OR “Neural Network (Computer)”)
19 TITLE-ABS-KEY (“Natural Language Processing” OR “Language Processing, Natural” OR “Language Processings, Natural” OR “Natural Language Processings” OR “Processing, Natural Language” OR “Processings, Natural Language” )
20 TITLE-ABS (“deep learn!” or “machine learn!” or “continuous learn!”)
21 TITLE-ABS (“neural network!”)
22 TITLE-ABS ((Artificial or Comput! or Machine) W/1 Intelligence)
23 TITLE-ABS (“Natural Language Processing!”)
24 TITLE-ABS (“Computer Vision!”)
25 or/17–24
26 16 and 25
27 (“systematic review” or “meta-analysis”)
28 TITLE-ABS-KEY ((“meta-analysis”) or (“systematic review” OR “Review, Systematic”) or (“systematic reviews as topic” OR “Systematic Review as Topic” OR “Reviews Systematic as Topic”) or (“meta-analysis as topic” OR “ Meta Analysis as Topic” OR “Data Pooling” OR “Data Poolings” OR “Overviews, Clinical Trial” OR “Clinical Trial Overviews” OR “Clinical Trial Overview” OR “Overview, Clinical Trial”) OR (“technology assessment, biomedical” OR “Biomedical Technology Assessment” OR “Technology Assessment, Health” OR “Assessment, Health Technology” OR “Assessments, Health Technology” OR “Health Technology Assessment” OR “Health Technology Assessments” OR “Technology Assessments, Health” OR “Assessment, Biomedical Technology” OR “Assessments, Biomedical Technology” OR “Biomedical Technology Assessments” OR “Technology Assessments, Biomedical” OR “Technology Assessment” OR “Assessment, Technology” OR “Assessments, Technology” OR “Technology Assessments” )OR (“network meta-analysis” OR “Meta-Analyses, Network” OR “Meta-Analysis, Network” OR “Network Meta Analysis” OR “Network Meta-Analyses” OR “Multiple Treatment Comparison Meta-Analysis” OR “Multiple Treatment Comparison Meta Analysis” OR “Mixed Treatment Meta-Analysis” OR “Meta-Analyses, Mixed Treatment” OR “Meta-Analysis, Mixed Treatment” OR “Mixed Treatment Meta Analysis” OR “Mixed Treatment Meta-Analyses”))
29 TITLE-ABS-KEY ((systematic! W/3 (review! or overview!)) or (methodologic! W/3 (review! or overview!)))
30 TITLE-ABS-KEY ((quantitative W/3 (review! or overview! or synthes!)) or (research W/3 (integrati! or overview!)))
31 TITLE-ABS-KEY ((integrative W/3 (review! or overview!)) or (collaborative W/3 (review! or overview!)) or (pool! W/3 analy!))
32 TITLE-ABS-KEY (“data synthes!” or “data extraction!” or “data abstraction!”)
33 TITLE-ABS-KEY (handsearch! or “hand search!”)
34 TITLE-ABS-KEY (“mantel haenszel” or peto or “der simonian” or dersimonian or fixed effect! or latin square!)
35 TITLE-ABS-KEY ((met AND analy!) or “metanaly!” or “technology assessment!” or HTA or HTAs or “technology overview!” or “technology appraisal!”)
36 TITLE-ABS-KEY (“meta regression!” or metaregression!)
37 TITLE-ABS-KEY (“meta-analy!” or “metaanaly!” or “systematic review!” or “biomedical technology assessment!” or “bio-medical technology assessment!”)
38 TITLE-ABS-KEY (comparative W/3 (efficacy or effectiveness))
39 TITLE-ABS-KEY (“outcomes research” or “relative effectiveness”)
40 TITLE-ABS-KEY ((indirect or “indirect treatment” or “mixed-treatment” or bayesian) W/3 comparison!)
41 TITLE-ABS-KEY (multi! W/3 treatment W/3 comparison!)
42 TITLE-ABS-KEY (mixed W/3 treatment W/3 (“meta-analy!” or “metaanaly!”))
43 TITLE-ABS-KEY (“umbrella review!”)
44 TITLE-ABS-KEY (multi! W/2 paramet! W/2 evidence W/2 synthesis)
45 TITLE-ABS-KEY (multiparamet! W/2 evidence W/2 synthesis)
46 TITLE-ABS-KEY (multi-paramet! W/2 evidence W/2 synthesis)
47 or/27–46
48 47 and 26
50 48 and (limit-to (pubyear, 2021) or limit-to (pubyear, 2020) or limit-to (pubyear, 2019) or limit-to (pubyear, 2018) or limit-to (pubyear, 2017) or limit-to (pubyear, 2016) or limit-to (pubyear, 2015)

**Table A1:** Datasets.

Type	Sub-Type	N
Database	Electronic Health Records	16
	Registry	14
	Administrative Database	2
	Other	3
Study	Retrospective Cohort	11
	Randomised Controlled Trial	10
	Prospective Cohort	10
	Cross-sectional	7
	Not Clearly Specified	4

**Table A2:** Methods *.

Category	Type	N
Machine Learning	Deep Learning	18
	Neural Network	16
	Ensemble	15
	Regression	14
	Decision Tree	13
	Instance-Based	11
	Bayesian	8
Artificial Intelligence	Clustering	6
	Dimensionality Reduction	5
	Rule System	4
	Regularisation	2

* The AI/ML algorithms were categorised using the framework adopted by Graili et al. [25] and proposed by Brownlee [26].
